# Recovery of carbon fiber-reinforced polymer waste using dimethylacetamide base on the resin swelling principle

**DOI:** 10.3389/fchem.2022.1050827

**Published:** 2022-11-01

**Authors:** Zixin Li, Mingfei Xing, Li Zhao, Zhan Li, Yaping Wang

**Affiliations:** ^1^ Institute of Resource and Environment, Henan Polytechnic University, Jiaozuo, China; ^2^ School of Surveying and Land Information Engineering, Henan Polytechnic University, Jiaozuo, China

**Keywords:** waste carbon fiber-reinforced polymer, dimethylacetamide, resin swelling, delamination, recovery

## Abstract

The mechanical recycling method of the carbon fiber-reinforced polymer (CFRP) has the advantages of simple process, less pollution and low cost, but only low utilization value of carbon fibers in powder or short fibers form can be obtained. To reduce the length and strength loss of the recycled carbon fibers, a novel and cost-effective dimethylacetamide (DMAC) swelling technique was developed to achieve rapid delamination of the CFRP laminates under mild conditions (120°C–160°C, 1 h). The corresponding swelling ratios and mass-loss rates of cured epoxy resin (CEP) were about 121.39%–157.39% and 0–0.69%, respectively. Excessive swelling of CEP in DMAC resulted in the cracking of the resin matrix between the adjacent carbon fiber layers. Thus the CFRP laminates were delaminated into soft single carbon fiber layers, which showed excellent cutting performance and reinforcing properties. The delamination products were cut into thin strips of different sizes and vacuum bag molded into new CFRP laminates. The flexural strength and tensile strength of the newly produced CFRP laminates were about 76.38%–90.98% and 94.61%–98.54% of the original CFRP laminates, respectively. More importantly, the chemical compositions of DMAC and CEP were unchanged during the physical swelling process. No organic pollutants (caused by resin degradation) were generated. And the used DMAC can be easily recycled by filtration. Therefore, this study provides a strategy for low-cost and high-valued recycling of CFRP waste.

## 1 Introduction

Advanced carbon fiber-reinforced polymer (CFRP) composites can be widely applied to new energy equipment (e.g., high-pressure vessel and wind turbine), lightweight transport facilities (aircraft and vehicle), and sporting tools (fishing rod and tennis racket) because of their low specific gravity, high strength, corrosion resistance, and good thermal stability (Lee et al., 2020; Zhang et al., 2020; Sun et al., 2022). However, numerous waste CFRP composites may be produced during the production and utilization processes. The generation of CFRP wastes reached 62000 tons in 2020 (Wei and Hadigheh, 2022). And the global waste CFRP composite output is estimated to be around nearly 500000 tons by 2050 (Lefeuvre et al., 2017). Improper disposal of CFRP waste may take up a substantial portion of land and contaminate the environment. Carbon fiber manufacturing is an example of a high-consumption industry (Dér et al., 2021). Approximately 198–595 MJ energy is consumed in the production of 1 kg of raw carbon fibers, while the greenhouse gas emissions are 31 kg carbon dioxide equivalent (Li et al., 2016; Meng et al., 2018). Recycling waste CFRP can effectively reduce energy consumption and carbon emission, lower the CFRP preparation cost, and promote the application in people’s daily lives.

Cured epoxy resins (CEP), which are typical thermosetting resins, are commonly used as CFRP structure matrices because they provide exceptional mechanical properties and corrosion-resistance to CFRP (Mustata and Tudorachi, 2017; Hanaoka et al., 2021; Wei et al., 2022). However, the excellent thermal and chemical stability of CEP makes the recycling of long and high-modulus carbon fibers from waste CFRP difficult (Hao et al., 2020). CFRP has been recycled *via* thermal processing (Oliveux et al., 2015; Hadigheh et al., 2021; Guo et al., 2022), chemical recycling (Kim et al., 2017; Kim et al., 2019; La Rosa et al., 2021; Hanaoka et al., 2022), and mechanical recycling (Li et al., 2016; Giorgini et al., 2020). The thermal recycling process is simple, but the high pyrolysis temperature (400°C–1000°C) results in high energy consumption and tensile strength loss of the recycled carbon fibers (about 15%–50%) (Zhu et al., 2019; Lu et al., 2022). Accordingly, some catalysts (Wu et al., 2019; Wu et al., 2022) (such as ZnCl_2_ and KCl) have been applied to reduce the pyrolysis temperature to ≤ 400°C and the tensile strength loss to ≤ 5%. Several solvents (strong acid, base and oxidant) with unique properties and catalysis have been used to degrade the resin matrix of CFRP by chemical recycling (Jiang et al., 2017; Liu et al., 2021; Huang et al., 2022). The reaction conditions of chemical recycling are relatively moderate, which aids in reducing the tensile strength loss of the recovered carbon fibers (Yu et al., 2016; Zhao et al., 2022). However, chemical methods also have disadvantages that must be addressed, such as large reagent consumption, harsh reaction conditions, and high equipment cost. Mechanical recovery methods can achieve the dissociation of resin matrix and carbon fiber by comminution or grinding process (Palmer et al., 2009). The processing cost and environmental impact of mechanical methods are lower than the traditional thermal processing and chemical recycling (Shuaib and Mativenga, 2016; Meng et al., 2017). However, soft carbon fibers are tightly bonded together by brittle CEP of glassy state in CFRP (Moosburger-Will et al., 2013; Tam et al., 2019). During the mechanical dissociation process of CFRP, long carbon fibers covered with a sheet of brittle CEP are easily broken into powder or staple fibers (Fonseca et al., 2020; Butenegro et al., 2021). The significant loss of carbon fiber length and strength (Gopalraj and Kärki, 2020) greatly limits the industrial application of mechanical recycling (Meng et al., 2018). Thus, the loss of carbon fiber strength and length must be mitigated to improve the reinforcing properties and recovery value of mechanical recovery products.

CEP is insoluble in common solvents because of its cross-linking structure. Nonetheless, CEP could swell in some organic solvents. The resin swelling phenomenon could benefit the penetration of catalysts so as to accelerate the reaction of degradation (Wang et al., 2015; Xing et al., 2021). In our previous study (Xing et al., 2021), an acetic acid swelling technology was developed as a pretreatment process of mechanical methods based on the theory of resin swelling. When CEP is fully swollen, its mechanical properties are significantly changed, transitioning from a glass state (brittle solid) to a highly elastic state (similar to rubber). Carbon fiber layers that contained in the CFRP laminates were delaminated from each other in acetic acid medium (160°C–220°C, 1 h). The soft delamination products were cut into various required shapes and hot pressed into new CFRP laminates. However, the acetic acid swelling technology also had some disadvantages: 1) the swelling temperature was higher which need to be further reduced to save energy. 2) Beside swelling effect, acetic acid also has a certain catalytic degradation effect on the resin. About 13.11%–23.33% of resin was degraded during the acetic acid swelling process result in the generation of many harmful organic pollutants (phenol and isopropyl-phenol, etc.). The new generated organic pollutants contained in the used acetic acid solution was also not conducive to the reuse of acetic acid solution.

In order to solve above problems, the swelling agent of acetic acid was replaced by DMAC which exhibited a better swelling effect on CEP. The delaminated temperature of CFRP could be obviously reduced from 160°C–220°C to 120°C–160°C in the DMAC swelling medium, and the resin was hardly degraded with less pollution in the recovery process (resin mass-loss rates were less than 0.70%). The flexural strength of the newly produced laminates (prepared by vacuum bag molding method) was 76.38%–90.98% of the original CFRP laminates. Accordingly, the energy consumption and environmental impact caused by resin degradation could be further reduced by the mild DMAC swelling method. In addition, DMAC has the characteristics of high thermal stability, and low corrosivity (Verma et al., 2017; Zhu et al., 2020). These features are conducive to recycling DMAC and reducing the corrosion of reaction equipment. Therefore, DMAC swelling technology was used in this study for low-cost and high-valued recycling of CFRP waste. The primary goals of this research are to 1) analyze the delamination properties of CFRP laminates in DMAC swelling media, 2) reveal the delamination process and mechanism of CFRP laminates in DMAC, 3) investigate the chemical composition changes of CEP and DMAC during the swelling process, and 4) examine the mechanical properties of the swelling CFRP laminates and the newly produced CFRP laminates.

## 2 Experimental section

### 2.1 Materials

The prepreg was WP-3011 (Weihai Guangwei Co., Ltd. China), a fabric composed of 60 wt% carbon fiber impregnated with 40 wt% uncured 6,508 epoxy resin as the matrix resin. The curing agent of 6,508 epoxy resin was dicyandiamide. The organic solvents used in this study were all analytically pure: DMAC (SCRC, ≥99.5%), DMSO (SCRC, ≥99.5%), DMF (SCRC, ≥99.5%), acetic acid (SCRC, ≥99.5%), ethyl alcohol (SCRC, ≥99.5%), dichloromethane (Kermel, ≥99.5%). KBr (Kermel, ≥99.9%).

### 2.2 Preparation of a carbon fiber-reinforced polymer laminate

An original CFRP laminate (8 cm × 10 cm×0.2 cm) was prepared from eight pieces of prepregs (8 cm × 10 cm×0.02 cm) by vacuum bag molding process. The curing temperature, curing time, and system pressure of the vacuum bag molding process were 120°C, 3 h, and 100 Pa, respectively.

### 2.3 Preparation of cured epoxy resin cube

CEP cube (1 cm × 1 cm×1 cm) was prepared for the better reveal the swelling effect on the resin volume expansion degree and resin cracking process*.* The liquid 6,508 epoxy resin was poured into a 1 cm cube silica gel mold. The silica gel mold was then placed into the curing oven for 3 h at 120°C. After the curing treatment, the solid CEP cube was removed from the silica gel mold.

### 2.4 Swelling recovery procedure of the carbon fiber-reinforced polymer laminates in dimethylacetamide

**FIGURE 1:**
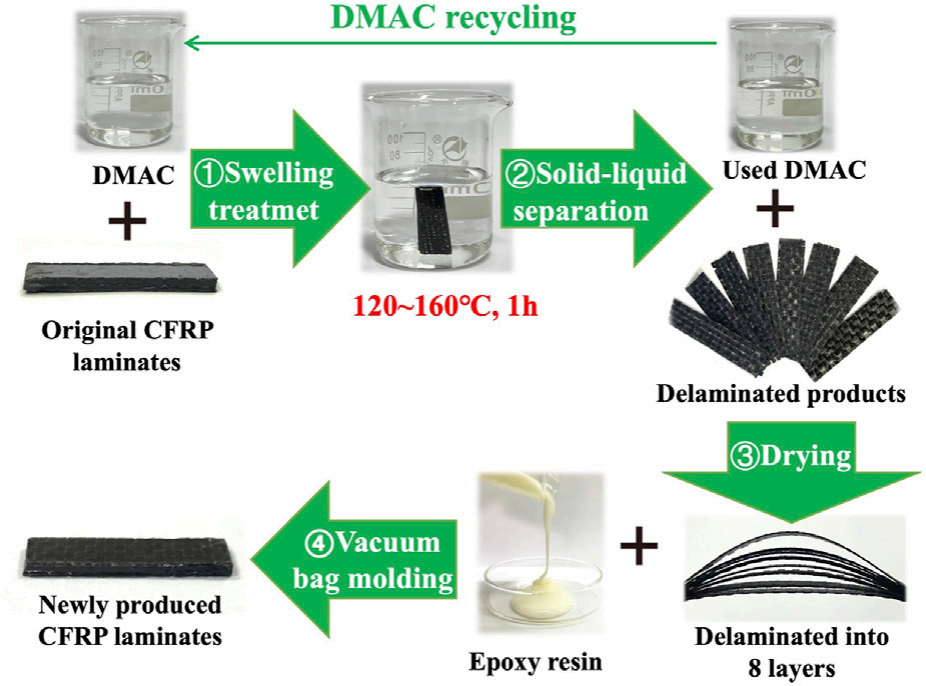
Delamination and recycling of the CFRP laminates by DMAC swelling technique.

Figure 1 displays the main swelling recovery process of the CFRP laminates in DMAC media. First, a CFRP laminate (2 g) or a *CEP cube* (0.5 g) was placed in a Teflon bottle (100 ml). Then, 45 ml of DMAC was placed into the Teflon bottle to ensure that the CFRP laminate or CEP cub*e* was immersed in the DMAC solution. Subsequently, the Teflon bottle was heated in a high temperature oven at 80°C–160°C (with an interval temperature of 20°C) and held for 0.5, 1, 2, 3, 4, and 6 h. The boiling point of DMAC at atmospheric pressure is 166.1°C, and the maximum swelling temperature was limited to 160°C. Finally, the Teflon bottle removed from the oven and allowed to cool to room temperature. The solid products (CFRP laminate or *CEP cube*) were collected by using a filtering method and dried at 105°C for further use.

The CFRP laminates were delaminated into eight pieces of soft carbon fiber layers in DMAC at 120–160°C within 1 h. Approximately 1 g of uncured epoxy resin was brushed on the surface of the delaminated products. Then, the delaminated products were vacuum bag molded into new CFRP laminates (the same curing condition as the original CFRP laminates). A part of the redundant liquid epoxy resin was expelled from the delaminated products during the vacuum bag molding process.

### 2.5 Dimethylacetamide recycling process

After the swelling treatment, the used DMAC obtained after solid–liquid separation was filtered with a filter pump (with a pumping speed of 30 L/min and a membrane diameter of 0.45 µm). The filtered DMAC could be recycled for CFRP swelling treatment.

### 2.6 Analysis

The resin swelling ratio was calculated using Eq. 1, while the resin mass-loss rate was computed using Eq. 2.

$$S\left(\%\right)=\frac{M_{1}-M_{2}}{M_{0}*M_{r}-\left(M_{0}-M_{2}\right)}\times 100\%$$ (1)

$$D\left(\%\right)=\frac{M_{0}-M_{2}}{M_{0}*M_{r}}\times 100\%$$ (2)

where *M*_0_ denotes the original CFRP laminate weight, *M*_1_ denotes the weight of the swelling CFRP laminate (contains a certain amount of DMAC), *M*_2_ represents the weight of the swelling CFRP laminate after drying treatment, and *M*_*r*_ represents the epoxy resin mass fraction of the original CFRP.

A three-point bending test was carried out for the measurement of the sample flexural strength at a loading speed of 0.2 cm/min, as shown in Eq. 3.

$$\sigma =\frac{3PL}{2bh^{2}}$$ (3)

where *P* denotes the load (N) measured; *L* denotes the span (mm); and *h* and *b* represent the thickness and width (mm), respectively.

A universal testing machine was used for the measurement of sample tensile strength at a loading speed of 1 cm/min, as shown in Eq. 4.

$$\sigma =\frac{F_{b}}{S_{o}}$$ (4)

where *F*_*b*_ is the maximum force sustained when the swelling product is broken (N), while *S*_*o*_ is the original cross-sectional area of the CFRP laminate (mm^2^).

A scanning electron microscope (SEM, Zeiss Supra 40, Germany) was utilized to observe the surface microstructure of the sample. Fourier transform infrared spectroscopy (FTIR, Shimadzu AIM-9000, Japan) was adopted to study the main chemical functional groups of the resin.

## 3 Results and discussion

### 3.1 Swelling properties of the carbon fiber-reinforced polymer in various solvents

**FIGURE 2:**
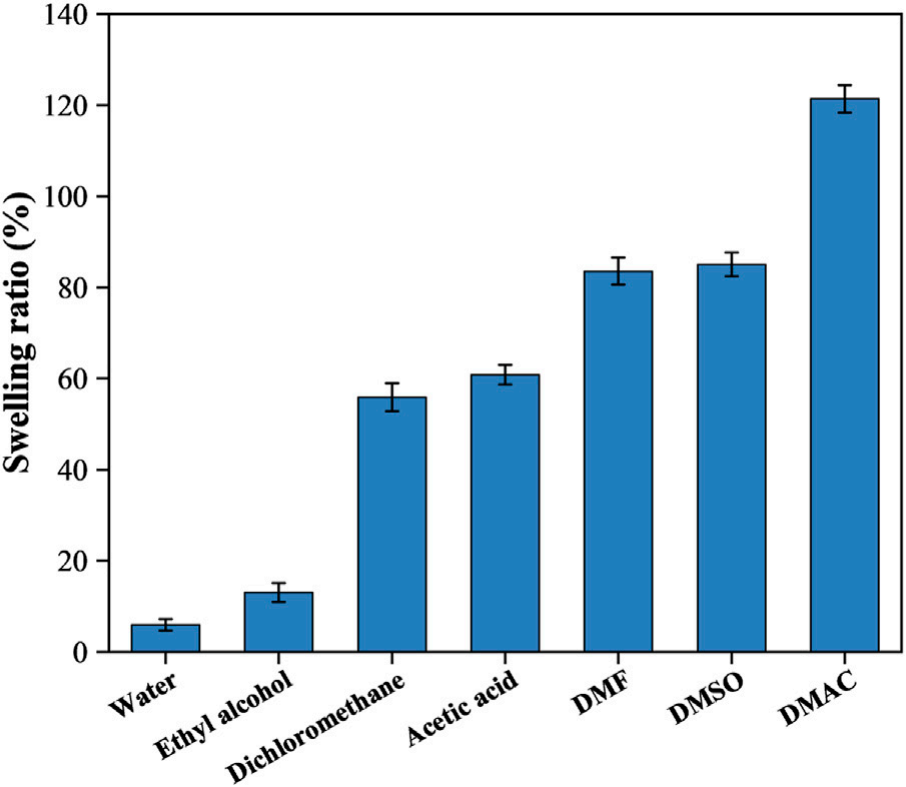
Swelling behavior of CFRP in various solvents at 120°C for 1 h.

The swelling properties of the CFRP in diverse solvents (120°C, 1 h) are shown in Figure 2. The swelling ability of water and ethyl alcohol is relatively low, and the swelling ratios were only about 5.96% and 13.08%, respectively. CFRP swelled the most in DMAC, with a swelling ratio of 121.39%. The swelling ratios of dichloromethane, acetic acid, DMF, and DMSO were 55.89%, 60.85%, 83.57%, and 85.02%, respectively. The selection of polymer swelling media was primarily based on the principle of similar polarity and solubility parameters (Zhu et al., 2018). Epoxy resin is a polar substance that contains several polar groups. DMF, DMSO, and DMAC are strongly aprotic polar solvents with an excellent swelling effect on epoxy resin (Verma et al., 2016; Tatariants et al., 2018; Han et al., 2019). Meanwhile, the solubility parameters of epoxy resin, DMF, DMSO, and DMAC are 19.85–20.46, 24.86, 26.68, and 22.77 MPa^1/2^, respectively (Hansen, 2007). The solubility parameters of DMAC and epoxy resin were close in value among the three solvents. Thus, DMAC was selected as the sweller in this study because it shows an excellent swelling effect on CEP. In addition, DMAC is an important solvent which is widely used in pharmaceutical, chemical applications and organic synthesis for its excellent thermal stability and low corrosivity. (Le Bras and Muzart, 2017; Gao et al., 2021).

**FIGURE 3:**
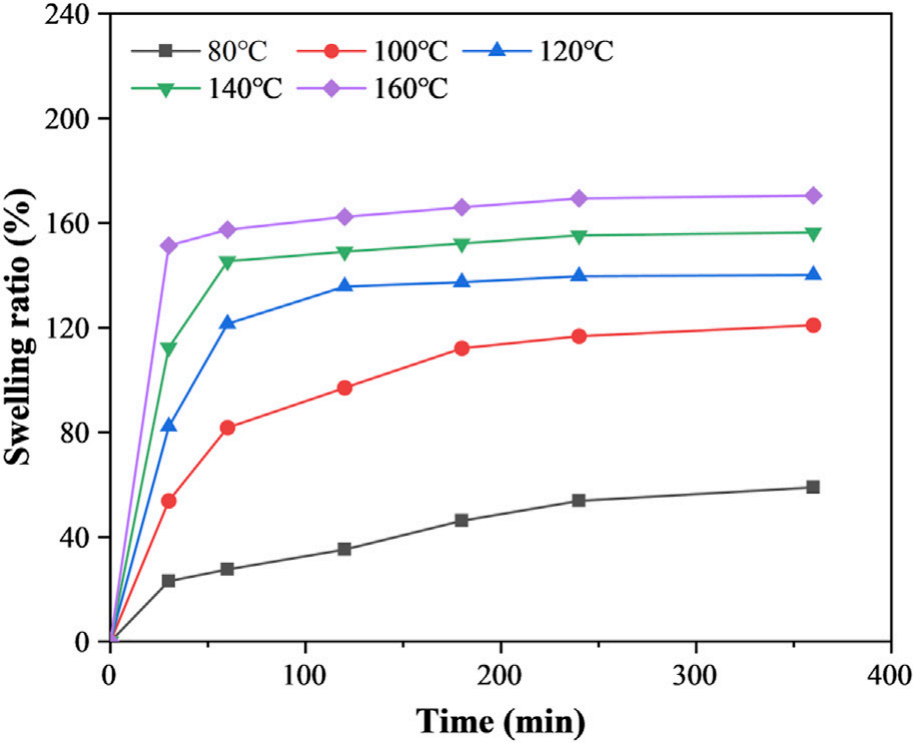
Swelling curves of CFRP in DMAC at different temperatures.

Figure 3 shows the swelling curves of CFRP in DMAC at different temperatures. The swelling ratio of CFRP increased along with the temperature enhancement and the time extension. A substantial amount of time was required to reach the swelling equilibrium at a low temperature (≤100°C). The swelling ratio at 100°C was only 120.93% when the swelling time was 6 h. However, the swelling ratio rapidly increased in the first hour and gradually reached the swelling equilibrium above 120°C. The swelling ratios were about 121.39%–157.39% at 120°C–160°C within 1 h. Meanwhile, the mass-loss rates of resin at 120°C, 140°C, and 160°C within 1 h were only 0%, 0.03% and 0.69%, respectively. This phenomenon indicated that CEP was stable in mild DMAC swelling media.

### 3.2 Delaminating characteristic of carbon fiber-reinforced polymer in dimethylacetamide

**FIGURE 4:**
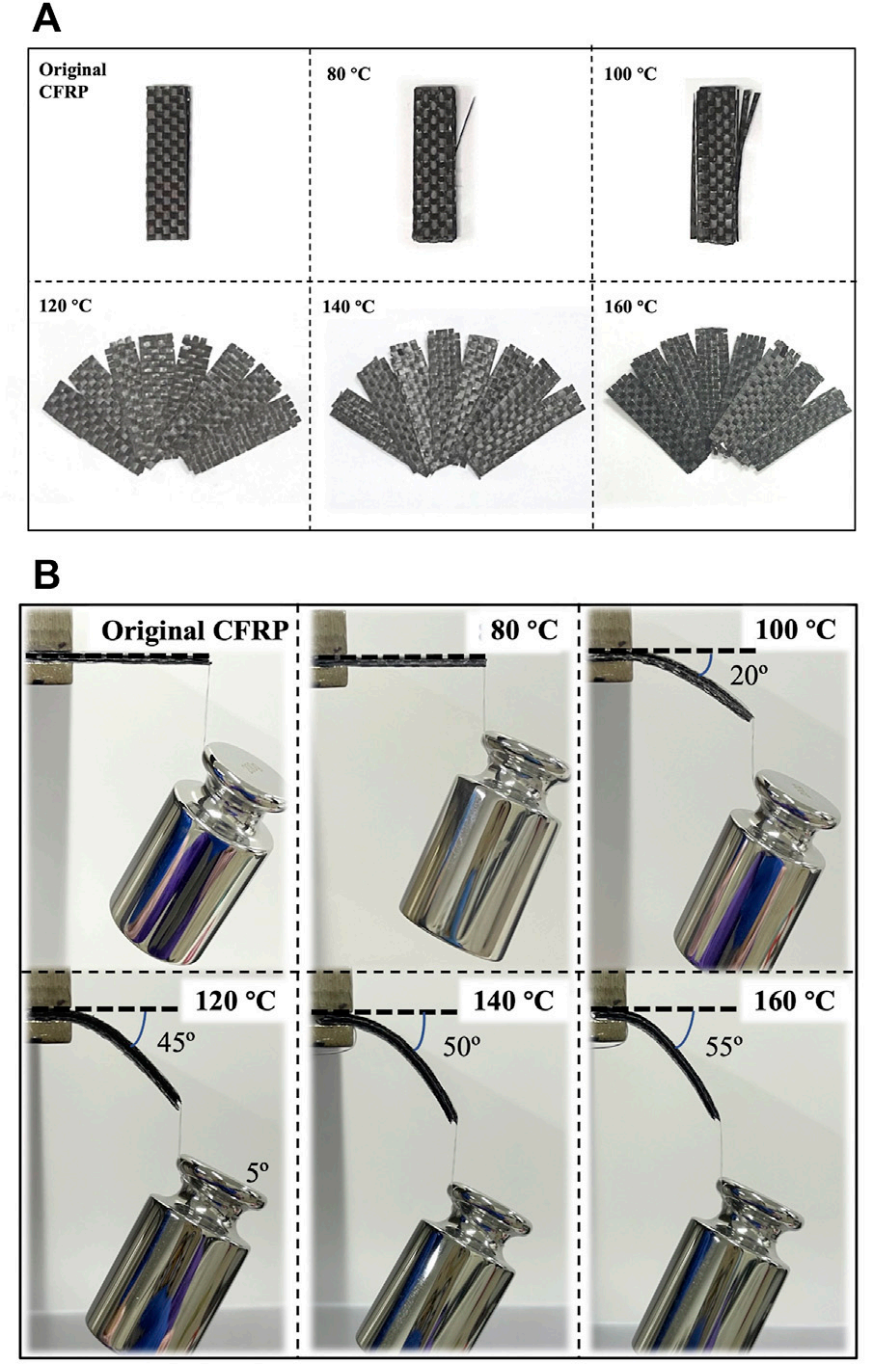
**(A)** CFRP laminates treated in DMAC at 80°C–160°C for 1 h and **(B)** corresponding load deformation comparison.

Figure 4A presents the images of the CFRP laminates treated in DMAC at 80°C–160°C for 1 h. Figure 4B shows the corresponding load–deformation comparison. Eight layers of carbon fibers were closely bonded together in the untreated CFRP laminates, and the stiff CFRP had no bending deformation under the load action. Part of the CFRP layers located on the CFRP surface began to peel off at 100°C, and the CFRP laminate was slightly bent under the load action. Above 120°C, the CFRP laminate began to delaminate into multiple single carbon fiber layers. Meanwhile, the CFRP laminate was bent under the load action. The experimental results indicated that the glassy state (brittle solid) of CEP could be changed into a highly elastic form (like rubber) by the CEP swelling phenomenon, which is helpful in improving the mechanical cutting performance of the CFRP laminates. The cross-linked segment movement of the glassy state CEP is frozen at low temperature, and only small-sized vibration and short-range rotational motion with low energy exist (Mijović and Zhang, 2004). The CEP obtained enough thermal energy and absorbed substantial DMAC as the swelling temperature increased, which gradually strengthened the interchain motion (Adamson, 1980). Therefore, the soft single carbon fiber layer could be cut into various required shapes.

**FIGURE 5:**
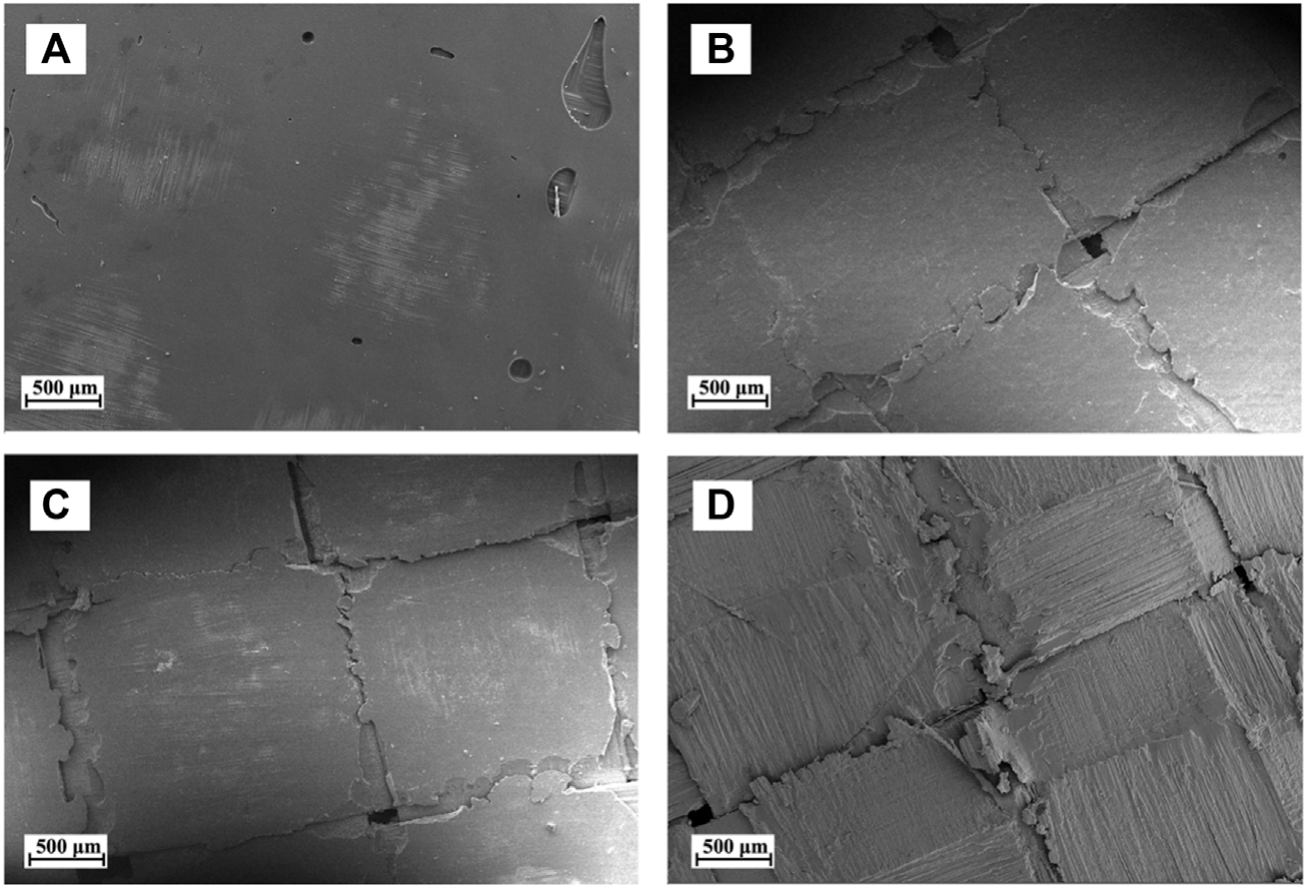
Surface morphology of the CFRP laminates treated under different temperatures: **(A)** original CFRP laminates, **(B)** 80°C, **(C)** 100°C, and **(D)** 140°C.

**FIGURE 6:**
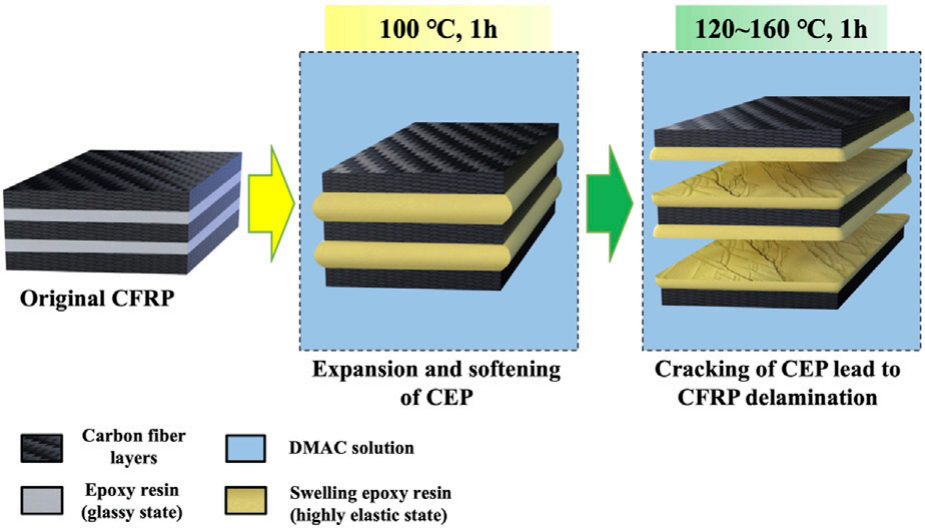
Possible delamination process of CFRP in DMAC.

The surface morphology of the CFRP laminates treated at different temperatures is shown in Figure 5. Figure 5A represents the micrographs of the original CFRP laminates. The surface of the untreated CFRP laminates was smooth and devoid of cracks. When the temperature was increased to 80°C–100°C, the volume of resin gradually expanded, resulting in the formation of many cracks at the intersection of longitude and latitude of the carbon fiber fabric, as shown in Figures 5B,C. These cracks might reduce the bonding effect between carbon fibers and influence the mechanical properties of the CFRP laminates. At 140°C, considerable CEP fell off the CFRP surface, and the carbon fiber layer was exposed, as shown in Figure 5D. The delamination phenomenon of CFRP was mainly attributable to the volume expansion and fragmentation of resin that distributed on the surface of the adjacent carbon fiber layers. Thus, sandwich model was used to approximate simulation of the swelling and delamination process of CFRP in DMAC (as shown in Figure 6).

### 3.3 Potential rapid swelling process and mechanism of cured epoxy resin in dimethylacetamide

**FIGURE 7:**
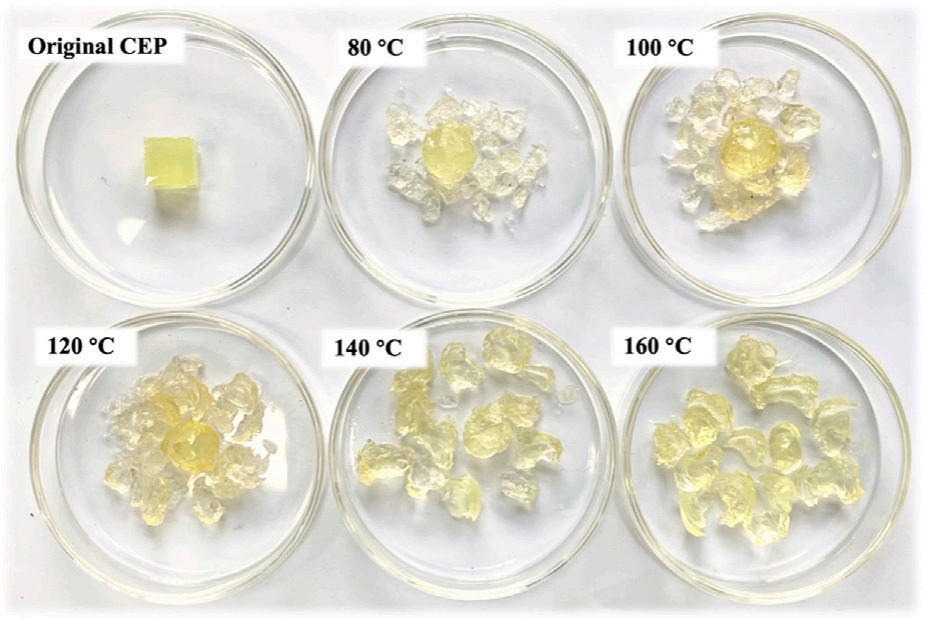
Images of the swelling products of the CEP cube treated at different temperatures.

Figure 7 shows the swelling products of the CEP cube obtained at different temperatures (80°C–160°C) when the swelling time and solid–liquid mass ratio of CEP/DMAC were fixed at 1 h and 1:25, respectively. The original CEP was a light yellow cube with a length of 10 mm. After the swelling treatment in DMAC at 80°C, only the CEP located on the cube surface cracked into small pieces and peeled off from the cube surface. This phenomenon confirmed that CEP could be fully swelled in DMAC under mild conditions. The volume expansion of resin was increased at a higher temperature, and the resin matrix eventually cracked into small pieces at 160°C. In addition, DMAC could quickly penetrate the inner part of the CEP cube along the cracks to further accelerate the resin swelling process.

**FIGURE 8:**
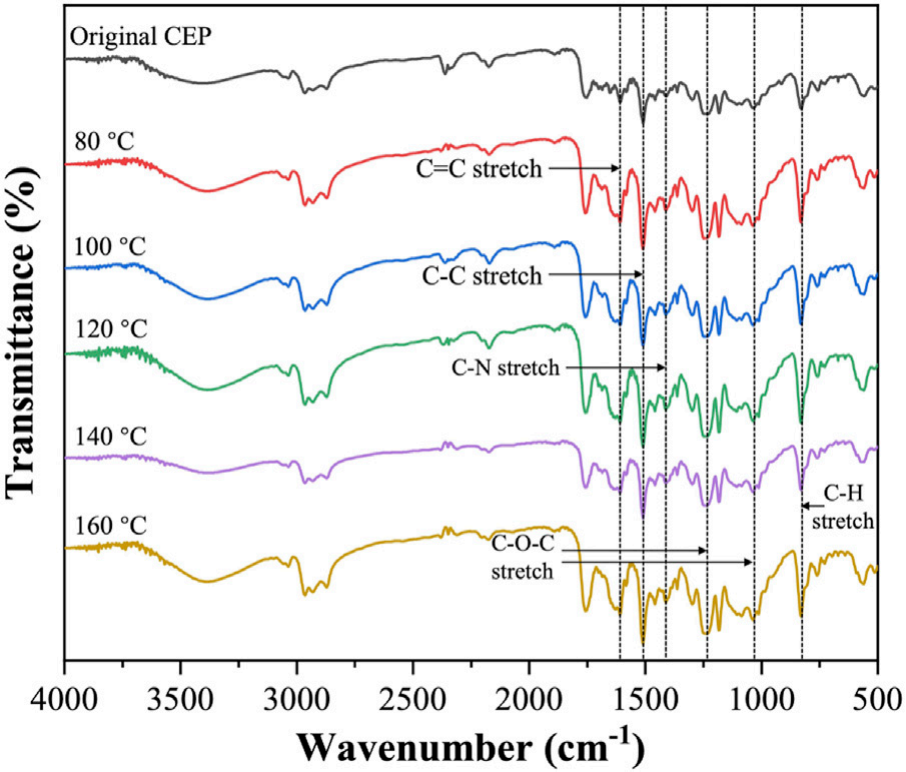
FTIR spectra of original CEP and swelling CEP treated at 80°C, 100°C, 120°C, 140°C, and 160°C.

Figure 8 shows the FTIR spectra of the CEP cube treated under different temperatures (80–160°C, 1 h). The peak intensity of the C–H stretch (830 cm^−1^), C–O–C stretch (1031 and 1246 cm^−1^), C–N stretch (1420 cm^−1^), C–C stretch (1508 cm^−1^), and C=C stretch (1607 cm^−1^) had no noticeable change with the increment of the swelling temperature (Guelachvili and Rao, 1986). This phenomenon indicated that the fragmentation of the resin matrix in DMAC was mainly attributed to the excessive expansion of the resin volume rather than the chemical degradation of CEP.

**FIGURE 9:**
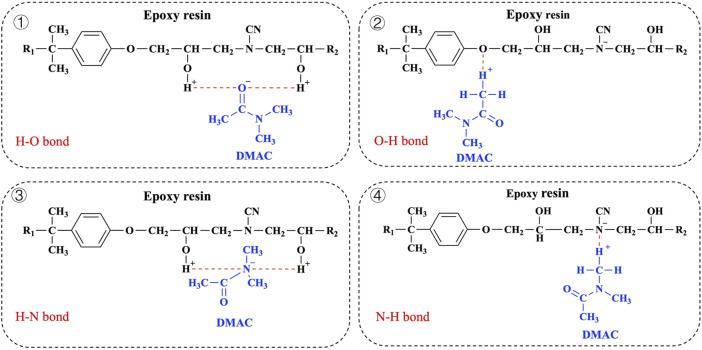
Possible swelling mechanism of CEP in DMAC media.

DMAC is a strongly nonprotonic polar solvent with an amide bond (–CO–NH–) in its molecular structure that can form hydrogen bonding interactions with numerous polar molecules (Verma et al., 2017; Pei et al., 2021). Meanwhile, epoxy resin as a polar material also contains many polar groups. The hydrogen bond acceptors of the resin segment include the unreacted epoxy group in the network structure, the O atom in the ether bond, the N atom on the tertiary amine, and the OH group. Specifically, the molecular structures of DMAC and epoxy resin (dicyandiamide cured bisphenol A epoxy resin) that can be used as hydrogen donors include the unreacted OH group of the resin network structure and the H atom of–CH_3_ contained in DMAC. The H acceptors include the O atom of C=O contained in DMAC, the N atom of DMAC, the On atom in the ether bond of the resin, and the N atom on tertiary amine atoms. The possible forms of hydrogen bond between CEP and DMAC are shown in Figure 9 (Zhu et al., 2013; Verma et al., 2017). The swelling mechanism can be explained by the hydrogen bonds present in the swelling system. However, the specific mechanism needs further study.

### 3.4 Mechanical properties of the carbon fiber-reinforced polymer swelling products

**FIGURE 10:**
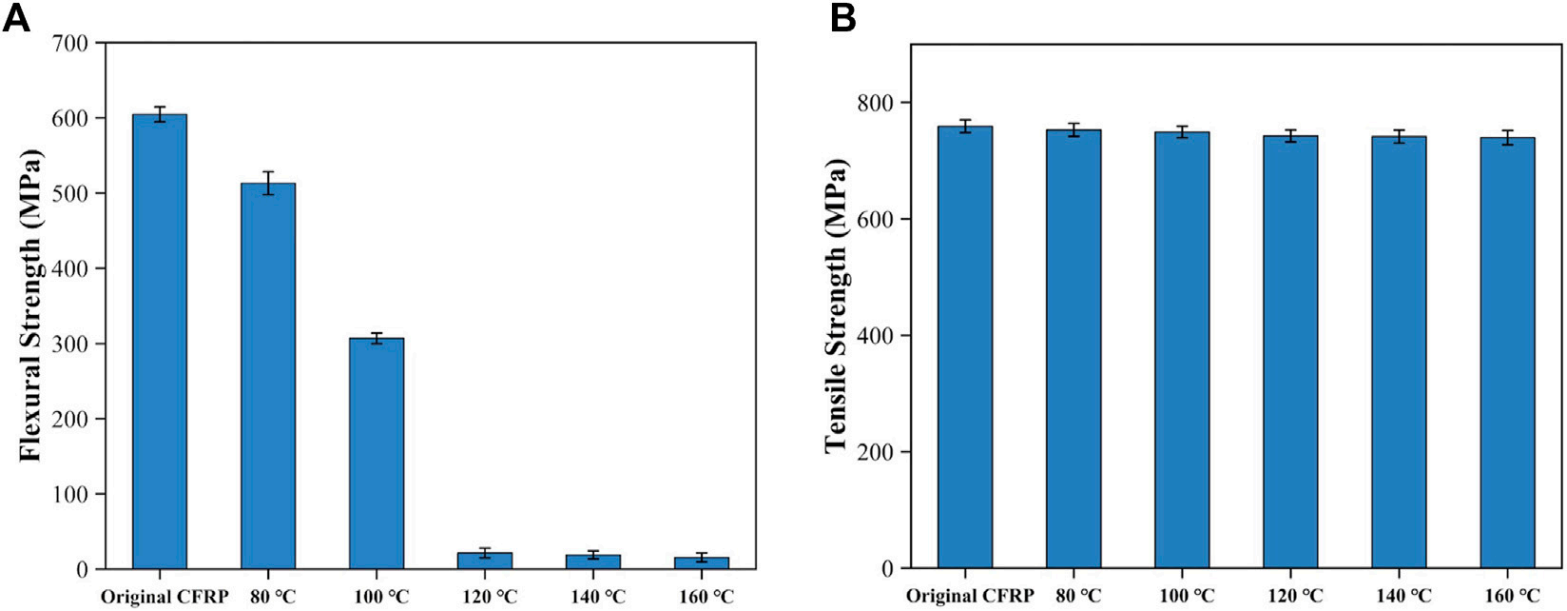
Mechanical properties of the swelling products: **(A)** flexural strength and **(B)** tensile strength.

Figure 10A displays the flexural strength of the swelled CFRP laminates after drying treatment. The original CFRP laminates had a high flexural strength (about 604.61 MPa). After the swelling treatment, the flexural strength quickly reduced from 513.07 MPa to 306.99 MPa as the swelling temperature rose from 80°C to 100°C. Above 120°C, eight layer of carbon fiber fabrics that contained in the CFRP laminates were delaminated from each other, and the flexural strength was significantly reduced to less than 22 MPa. The reduction of flexural strength was mainly attributed to the fragmentation phenomenon of CEP, which could reduce the adhesion degree between the adjacent carbon fiber fabric. Figure 10B shows the tensile strength of the single-layer CFRP treated at different temperature. The tensile strength of the single-layer CFRP was slightly decreased from 752.91 MPa MPa to 739.59 MPa as the swelling temperature rose from 80°C to 160°C. And the tensile strength was 97.44%–99.19% of the original single-layer CFRP (759.05 MPa). Accordingly, the mild swelling system has a minimal influence on the mechanical properties of the carbon fibers.

### 3.5 Mechanical properties of the newly produced carbon fiber-reinforced polymer laminates

**FIGURE 11:**
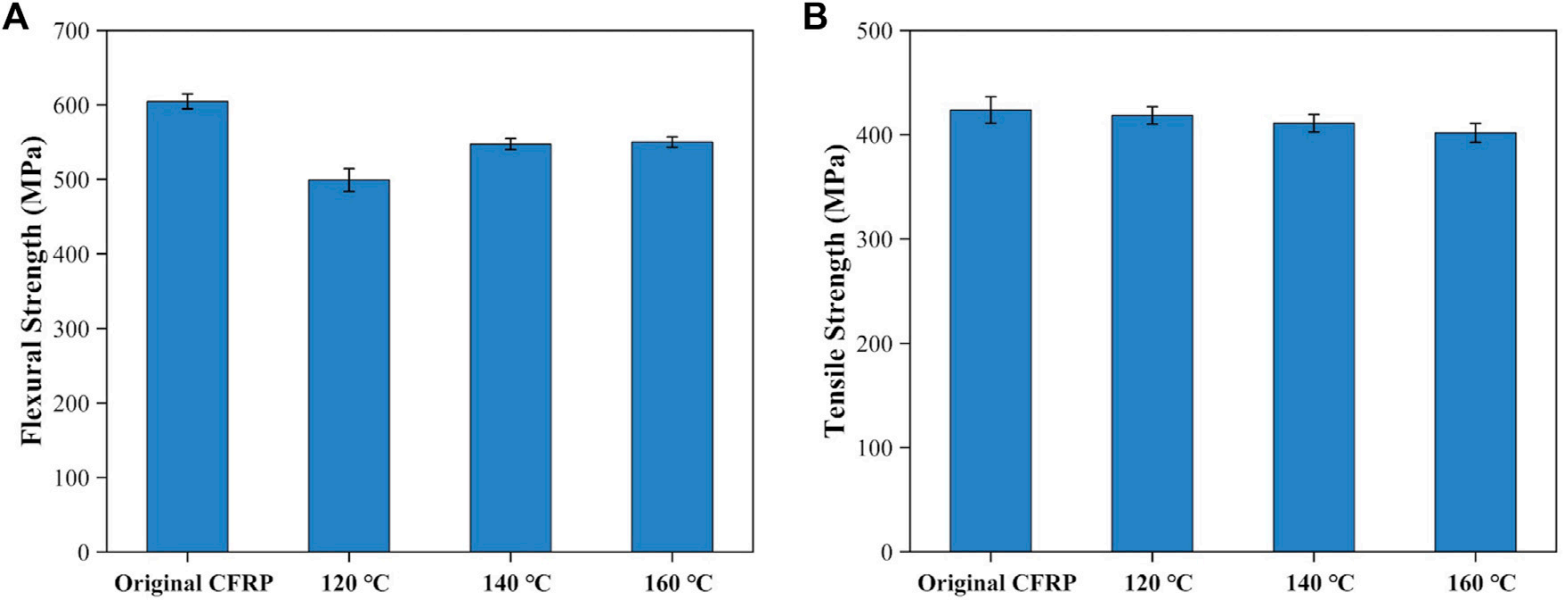
Mechanical properties of the newly produced CFRP laminates: **(A)** flexural strength and **(B)** tensile strength.

The dried delamination products were vacuum bag molded again to create new CFRP laminates. The flexural strength and tensile strength of the newly produced CFRP laminates were shown in Figure 11. The newly produced CFRP laminates’ flexural strength was approximately 76.38%–90.98% that of the untreated CFRP laminates as shown in Figure 11A. Then the flexural strength increased from 499.21 MPa to 547.41 MPa as swelling temperatures increased from 120°C to 140°C. Finally, the flexural strength reached the maximum value of 550.07 MPa at 160°C. The possible reason was that the degree of resin swelling increased under elevated swelling temperature leading to the increment of cracks’ size and number and more resin was fell off from the carbon fiber layer’s surface. Under the vacuum condition, the infusion of the liquid epoxy resins could fill these large cracks and tightly bonded to the surface of the carbon fiber layer to revive the CFRP laminates’ flexural strength (Xing et al., 2021). Figure 11B shows the tensile strength of the new produced CFRP laminates. The tensile strength of the newly produced CFRP laminates was 401.78 MPa–418.49 MPa, which was 94.61%–98.54% of the untreated CFRP laminates (424.68 MPa). This phenomenon suggested that the effect of DMAC swelling treatment on the mechanical properties of carbon fiber was insignificant.

**FIGURE 12:**
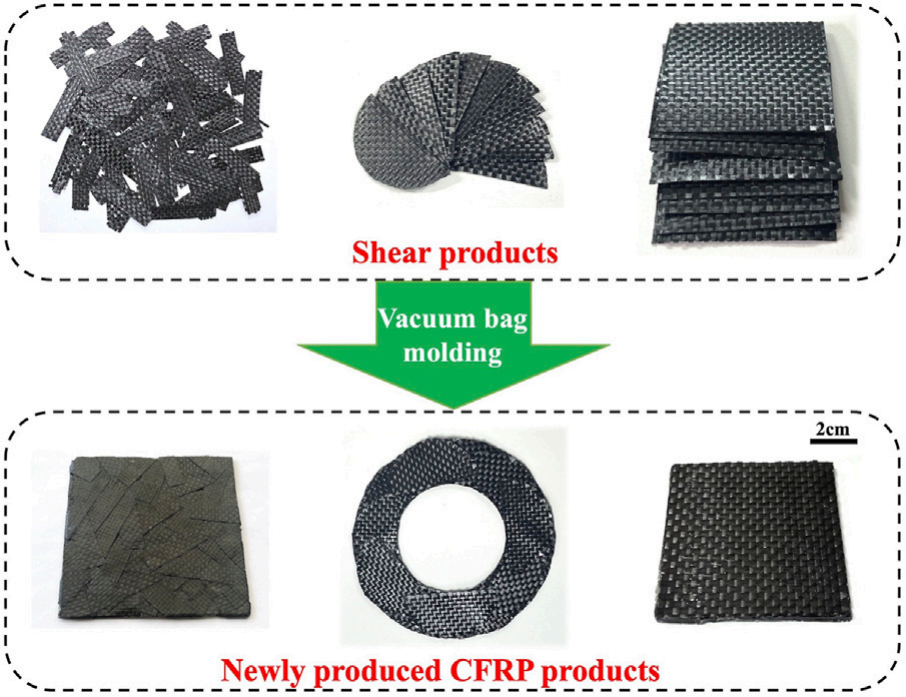
Different utilization methods for the CFRP delamination products.

The soft delaminated products could be easily cut into thin slices of various shapes in the practical application, as shown in Figure 12. Furthermore, the shear products could be prepared into different CFRP products *via* a vacuum bag molding process. The newly produced CFRP products could be used for general load-bearing components, such as carbon fiber seats, bicycle wheels, and fishing platforms. However, the mechanical properties of the newly produced products were also related to the size of the shear product, the kind and amount of the resin matrix, and the molding type. In addition, CEP contained in CFRP was not degraded during the swelling process and was transferred into the newly produced CFRP products. Thus, the DMAC swelling technique could realize the overall utilization of waste CFRP.

### 3.6 Dimethylacetamide recycling

**FIGURE 13:**
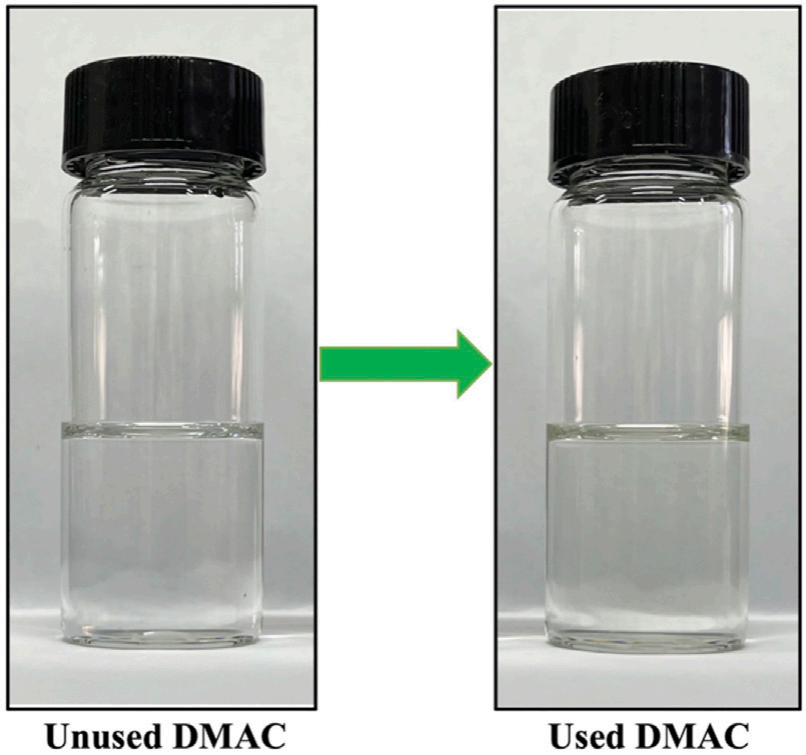
Images of the unused and used DMAC (filtrated).

**FIGURE 14:**
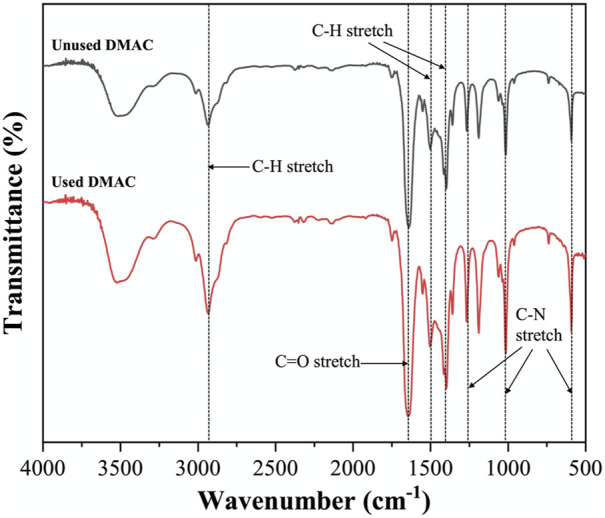
FTIR spectra of the unused DMAC and used DMAC (filtrated).

Figures 13, 14 show the corresponding FTIR spectra of the unused and used DMAC (filtrated). No significant change can be observed in the DMAC color before and after swelling treatment in Figure 13. DMAC contains several chemical bonds, such as C–H bond (3000–2850 cm^−1^), C–N bond (1250, 1020, and 657 cm^−1^), and C=O bond (1670 cm^−1^) (Guelachvili and Rao, 1986; Verma et al., 2017). No significant change was observed between the FTIR spectra of the unused and used DMAC. The FTIR result validated that DMAC has good thermal and chemical stability under mild swelling conditions. The recycling of DMAC can be achieved by simple filtration using the Millipore filter (Nylon 66, 0.22 μm), which is conducive to reducing the treatment cost and environmental pollution. In addition, DMAC is flammable liquid and toxic gases may be generated when burning. Thus, temperature control (<160°C) is required in the actual production process to prevent combustion or thermal decomposition of DMAC. And other environmentally-friendly swelling solvent should be explored in future research.

## 4 Conclusion

The DMAC swelling technology could realize rapid softening and delamination of the CFRP laminates under mild conditions (120°C–160°C, 1 h) due to the excellent swelling effect of DMAC on CEP. The corresponding swelling ratios and mass-loss rates of CEP were about 121.39%–157.39% and 0–0.69%, respectively. The overexpansion of the CEP volume resulted in the fragmentation of CEP between the adjacent carbon fiber layers of CFRP, and CFRP was delaminated into soft single layers. The delaminated product’s flexural strength was only about 2.55%–3.55% of the original CFRP laminates, while the tensile strength minimally decreased (≥97.44%). The obtained soft carbon fiber layer could be easily cut into various required shapes. Then, the shear products were dried and mixed with a small amount of liquid epoxy resin to obtain a new CFRP laminate by using a vacuum bag molding process. The cracks in the shear products can be filled with epoxy resin under the vacuum environment. Thus, the flexural strength and tensile strength of the newly produced CFRP laminates were about 76.38%–90.98% and 94.61%–98.54% of the original CFRP laminates, respectively. After the swelling process, the chemical composition of DMAC was unchanged, which was conducive to realizing the recycling of DMAC. Therefore, this study may provide a novel and cost-effective approach for CFRP recycling.

## Data Availability

The original contributions presented in the study are included in the article/supplementary material, further inquiries can be directed to the corresponding author.
